# Increased Risk of New-Onset Rheumatoid Arthritis Among Osteoarthritis Patients Received Total Knee Arthroplasty: a global federated health network analysis

**DOI:** 10.7150/ijms.93457

**Published:** 2024-04-08

**Authors:** Zong-Han Lin, Hui-Chin Chang, Yu-Lun Wu, Shuo-Yan Gau

**Affiliations:** 1 Department of Medical Education and Research, Kaohsiung Veterans General Hospital, Kaohsiung, Taiwan.; 2 Evidence-based Medicine Center, Chung Shan Medical University Hospital, Taichung, Taiwan.; 3 Library, Chung Shan Medical University Hospital, Taichung, Taiwan.; 4 School of Medicine, Chung Shan Medical University, Taichung, Taiwan.; 5 Department of Neurosurgery, Kaohsiung Veterans General Hospital, Kaohsiung, Taiwan.; 6 School of Medicine, College of Medicine, National Sun Yat-sen University, Kaohsiung, Taiwan.; 7 Department of Medical Education, Kaohsiung Chang Gung Memorial Hospital, Kaohsiung, Taiwan.; 8 Orthopedics Department, Chi-Mei Medical Center, Tainan, Taiwan.

**Keywords:** osteoarthritis, cohort, epidemiology, electronic medical records, rheumatoid arthritis, real world study

## Abstract

**Background:** Complications of total knee arthroplasty (TKA) had been widely discussed. However, whether TKA influence risk of rheumatoid arthritis (RA) in osteoarthritis patients remained uncertain. We intend to evaluate the risk of RA in osteoarthritis patients underwent TKA.

**Methods:** In this retrospective cohort study, data was retrieved from the US collaborative networks in TriNetX research network. Within the study period between 2005 and 2017, osteoarthritis patients underwent TKA were enrolled as case cohort whereas osteoarthritis patients never underwent TKA were enrolled as control cohort. Covariates were matched via propensity score matching. Risk of RA in TKA patients were valuated under various follow-up time and sensitivity models.

**Results:** Under 1-year, 3-year and 5-year of follow-up, TKA patients were associated with significantly elevated risk of RA, especially under 1-year follow-up (HR=1.74; 95% CI, 1.39-2.18). Subgroup analysis demonstrated a significant increase in the risk of RA following TKA in the female subgroup (HR=1.42; 95% CI, 1.24-1.63), the subgroup aged 18-64 years (HR=1.48; 95% CI, 1.11-1.97), and the subgroup aged greater than 65 years old (HR=1.38; 95% CI, 1.21-1.58) based on 5-year follow-up.

**Conclusion:** Clinicians should be concerned about uncharted association between TKA and RA reported our current study. Additional prospective studies and in-depth mechanistic inquiries were warranted to determine the causation.

## Introduction

Rheumatoid arthritis (RA) is an autoimmune ailment defined by long-term symmetrical inflammatory peripheral polyarthritis^1^. It invariably leads to joint destruction through cartilage and bone erosion^2^,^3^. If left untreated, RA can result in functional impairment, hindrance in daily tasks, and challenges in maintaining employment^4^-^8^. The uncontrolled inflammation associated with RA may contribute to adverse long-term outcomes and elevate the risk of various health issues, including cardiovascular disease^9^,^10^, interstitial lung disease^11^, peripheral vascular disease^12^-^14^, osteoporosis^15^ and osteoarthritis^16^. Epidemiologic and related studies have identified a range of genetic^17^,^18^, demographic^19^,^20^, lifestyle^21^ as risk factors for RA. The substantial impact of RA on healthcare systems and society is evident through regular medical visits, prolonged disease-modifying antirheumatic drug (DMARD) treatments, heightened disability and work loss, diminished quality of life, and premature mortality. These factors contribute to an estimated annual social cost exceeding $39 billion^22^. Given the intricate nature of RA, there is a compelling need to delve into the multifaceted origins and consequences of this chronic autoimmune disease.

Concurrently, Total Knee Arthroplasty (TKA) has emerged as a pivotal intervention for joint-related pathologies^23^, providing relief and enhanced functionality for numerous individuals. However, recent investigations have illuminated the interactive nexus between implants and autoimmune diseases. Research indicates an association between breast implants and an increased risk of RA^24^,^25^, with analogous effects potentially extending to metal implants^26^-^28^. Metal implants may instigate autoimmune disorders, and individuals with autoimmune disorders may experience more frequent allergic reactions to metal implants^29^, which, in turn, could contribute to the progression of connective tissue disorders^29^. Notably, there have been instances of systemic lupus erythematosus in patients sensitized to molybdenum^30^. Moreover, the use of bone cement has been linked to depigmentation^31^.

We have noted the intricate relationship between TKA as a crucial treatment for osteoarthritis and the potential of implants to trigger autoimmune diseases^32^. However, a comprehensive understanding of this phenomenon is lacking due to the absence of large-scale studies. To address this knowledge gap, we conducted a retrospective study utilizing the TriNetX - U.S. Collaborative Network. The primary objective of our study was to leverage a substantial database to investigate whether knee replacement surgery contributes to an increased risk of RA. By exploring the association between TKA and the onset of RA, our aim is to provide valuable insights into the complex interplay between orthopedic interventions and autoimmune disorders. We anticipate that our findings will inform future clinical practices, facilitating the optimization of patient care post-joint replacement surgeries.

## Methods and Materials

This study was performed based on a retrospective cohort design. The TriNetX research network, a global-federated electronic health record database, was applied as the data source of this study. De-identified, prospectively updated electronic health records from patients in the collaborative healthcare organizations (HCOs) were available in the TriNetX research network for statistical analyses. In the current design, a subset of TriNetX, the US collaborative network, has been utilized. This subset retrieved the health data from 60 HCOs in the United States, and contained greater than 80 million patients' data. This subset of TriNetX was widely applied in epidemiological studies in various fields of clinical investigation^33^-^35^.

Patients with visit record and being diagnosed of osteoarthritis at the period between January 2005 and December 2017 were enrolled for further analyses. Since the database was prospectively updated, we ensured that each participant could be applied with a follow-up time greater than five years. For the enrolled osteoarthritis patients, those who underwent TKA were identified as the TKA cohort. Osteoarthritis patients with no TKA record before the index date were identified as non-TKA control cohort. For both cohorts, any individuals who being diagnosed of autoimmune diseases (including ankylosing spondylitis, dermatopolymyositis, Sjögren syndrome, systemic lupus erythematosus, psoriasis. ulcerative colitis, Crohn's disease, rheumatoid arthritis) or any cancers would not be included for further analyses. Moreover, people less than 18 years old or those who died before index date would be excluded from the study population. Applied administrative codes were reported in detail in **Table S1**.

In each analysis evaluating hazard ratio, propensity score matching was performed to address the potential baseline difference between the TKA cohort and control cohort. In every instance of the propensity score matching process, a greedy-nearest neighbor algorithm was employed, integrating the standard deviations with a caliper width set at 0.1. In the main analysis, matched covariates included age, sex, race, obesity status, inflammatory status, comorbidities, comedication use, lifestyle, socioeconomic issues, medical utilization status. Sensitivity analyses were performed based on multiple propensity score matching algorithms to address potential bias caused by overmatching. Moreover, exclusion of incident outcome event has been performed based on different wash-out period to address bias caused by reversed causality. To further investigate the influence of incident rheumatoid arthritis in TKA patients, we performed stratification analysis based on populations in different age and sex. Cox regression method were performed in each sensitivity and stratification analysis to provide comparison of hazard ratio in developing further RA in the TKA and non-TKA cohorts.

The formal analysis has been performed via the Analytics function of TriNetX research network. Hazard ratio (HR) has been calculated in each analysis evaluating the future risk of rheumatoid arthritis in TKA group while comparing with controls, with the 95% confidence intervals (95% C.I.) presented with the HR to evaluate the significance of the result. While presenting the baseline characteristics before and after matching, standardized difference (SD) were calculated to represent the difference of each covariates between TKA group and control group. When the value of SD more than 0.1, the difference between two groups were regarded as significant.

## Results

### Baseline characteristics of patients with and without TKA

**Figure 1:**
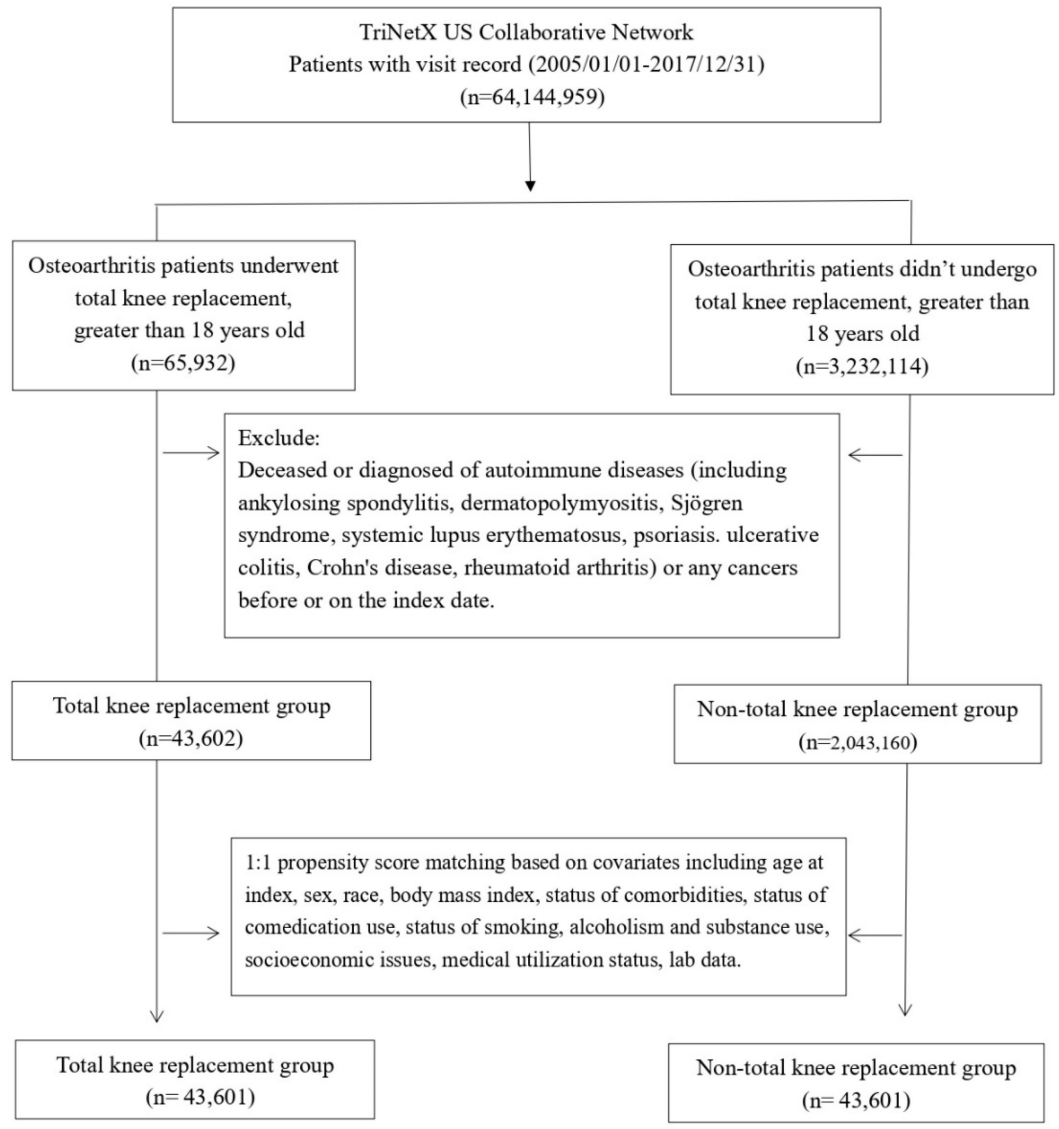
Flowchart of participant selection.

Patients diagnosed with OA were categorized into an experimental group (n=65,932) undergoing TKA and a control group (n=3,232,114) not receiving TKA. After excluding individuals with autoimmune diseases and cancer, a total of 43,602 patients were enrolled in the experimental group, and 2,043,160 patients were enrolled in the control group. Subsequently, employing 1:1 propensity score matching, 43,601 patients were included in the experimental group, and 43,601 patients were retained in the control group (**Figure 1**).

Following the matching process, there were no significant differences between patients who underwent TKA and unmatched patients regarding age, sex, race, and medication use (Glucocorticoids, Beta blockers), body mass index, and medical utilization status (**Table 1**). Nevertheless, the analysis of baseline characteristics revealed that the TKA group exhibited a higher percentage of patients with alcohol dependence, smoking, substance use, comorbidities, health hazards, and the use of antidepressants compared to the control group. Moreover, the proportion of C-reactive protein ≥ 3 (mg/L) in the TKA group was notably higher than that in the control group. In the baseline, the difference between erythrocyte sedimentation rate (ESR), antinuclear antibody (ANA), anti-cyclic citrullinated peptide antibody (anti-CCP) and rheumatoid factor (RF) did not present significant difference between TKA and non-TKA groups.

### Risk of RA in osteoarthritis patients underwent TKA

The hazard ratio estimation, conducted using the analysis function of the TriNetX research network, examined the association between various follow-up periods and the risk of RA. The results consistently demonstrated a positive trend overall (**Table 2**). Under 1-year, 3-year and 5-year of follow-up, TKA patients were associated with significantly elevated risk of RA, especially under 1-year follow-up (HR=1.74; 95% CI, 1.39-2.18). The incidence rate of RA in TKA group was 0.000008 person-day, and in non-TKA group, the incidence rate of RA was 0.000008 person-day (**Table S2**). Subgroup analysis demonstrated a significant increase in the risk of RA following TKA in female subgroup (HR=1.42; 95% CI, 1.24-1.63), the subgroup aged 18-64 years (HR=1.48; 95% CI, 1.11-1.97), and the subgroup aged >64 years (HR=1.38; 95% CI, 1.21-1.58) (**Table 3**).

### Sensitivity analyses

Various sensitivity analyses were conducted to explore the association between TKA and RA risk (**Table 4 and Table S3**). In the first set of analyses applying various propensity matching algorithms (**Table 4**), Model 1 involved matching age at index and sex through propensity score matching, resulting in an HR of 1.25 (95% CI, 1.11- 1.41). Model 2 matched age, sex, and comorbidities, yielding an HR of 1.23 (95% CI, 1.10- 1.38). Model 3 involved matching age, sex, substance use, and comedications, resulting in an HR of 1.25 (95% CI, 1.11-1.40). In the second set of analyses (**Table S3**), patients diagnosed of RA within one year, two years, and three years after TKA were excluded. The calculated HRs were 1.30 (95% CI, 1.15-1.47), 1.39 (95% CI, 1.21-1.59), and 1.46 (95% CI, 1.26-1.70), respectively. In order to evaluate the generalizability of the TKA-RA association, aside from osteoarthritis population, we performed an additional sensitivity analysis to evaluate the risk of RA in TKA patients while comparing with general population. We found that under 5-year-follow-up, the risk of TKA group with OA has 1.87-fold of risk in developing future RA (95% CI, 1.66-2.11). Under 1-year-follow-up period, the risk elevates to 2.76-fold higher than general population (95% CI, 2.13-3.58) (**Table S4**).

## Discussion

Findings of this study indicate a notable rise in the risk of subsequent RA in osteoarthritis patients who have undergone TKA compared to those who have not. Even with the consideration of multiple covariates, the observed association between post-TKA surgery and the reported heightened risk of RA persisted significantly in statistical models. Furthermore, the results retained their significance even after the exclusion of individuals diagnosed with RA within 1 to 3 years of surgery.

Studies have underscored a significant interplay between implants and autoimmune diseases. For instance, a case report documented a 24-year-old woman who manifested symptoms of systemic lupus erythematosus (SLE) following the implantation of a metal plate^30^. It was revealed that she exhibited delayed-type hypersensitivity to molybdenum, and her symptoms improved following the subsequent removal of the implant. Another case involved a 72-year-old woman who developed aseptic periprosthetic arthritis after TKA, suspected to be linked to metal release and cobalt sensitization from the artificial joint^36^. Additionally, a 23-year-old woman undergoing nickel-titanium chin implantation experienced a spectrum of symptoms, leading to a diagnosis of autoinflammatory syndrome, which could potentially be induced by adjuvants^37^.

A comprehensive retrospective study exploring the correlation between dental amalgam and arthritis, encompassing 86,305,425 weighted individuals in the exposed group, indicated a six-fold higher risk of arthritis compared to the control group^26^. Metals, with their potential to induce immunosuppression, immunotoxicity, or act as immune adjuvants, may incite allergy and autoimmunity, a phenomenon that has shown an escalating incidence^38^. Beyond metal implants, the utilization of intraoperative bone cements may also heighten the susceptibility to autoimmune diseases. Benzoyl Peroxide, a crucial constituent of bone cements, has been associated with conditions such as leukoplakia and allergic complications^31^. Moreover, individuals with autoimmune disorders exhibit an increased likelihood of experiencing allergic reactions to metal implants. Noteworthy is the observation of delayed-type hypersensitivity reactions to nickel, mercury, gold, palladium, titanium, or chromium in patients with connective tissue disorders like SLE and RA^39^. Chronic inflammatory reactions induced by metals can further exacerbate connective tissue disorders^29^.

Furthermore, C-reactive protein (CRP) is considered to be associated with systemic inflammation, and patients with osteoarthritis may have higher serum CRP levels, which are correlated with symptom severity 40. Similarly, elevated CRP levels can be observed in patients with rheumatoid arthritis (RA) and may vary with the severity of inflammation and synovitis 41. Untreated RA patients may also present with acute phase reactants; however, such findings are uncommon, and alternative diagnoses should be considered as a priority. Therefore, the influence of CRP levels on subsequent RA diagnosis is relatively minor^42^. Nonetheless, to minimize experimental bias resulting from differences in baseline inflammation, we matched patients with CRP levels ≥ 3 (mg/L) in our study. The results indicated that patients with high inflammatory indices constituted a lower proportion of the overall study population, with no significant differences observed between the two groups.

Joint replacement surgery, particularly hip and knee implants, has been linked to autoimmune diseases, particularly connective tissue diseases. A substantial body of evidence supports this association. A retrospective study reported that in post-implantation individuals, a heightened risk of developing autoimmune or connective tissue disease was observed^27^. Another large retrospective study in Denmark stated that compared with osteoarthritis patients who did not undergo joint replacement, those who underwent hip replacement had significantly higher rates of hospitalization for dryness and systemic lupus erythematosus. Patients undergoing knee replacement surgery had a significantly increased risk of hospitalization for RA, CTD, and dryness^43^. A small-scale case-control study found a increase in the incidence of undifferentiated connective tissue disease (UCTD) after exposure to silicone-free artificial joints, with the risk greater than 5-fold observed. Significant increase in the incidence of UCTD after exposure to orthopedic metallic fixation devices was also noted^32^. Exposure to silicone-containing devices (shunts and catheters) was observed to be significantly associated with CTD. In light of this compelling evidence, our study aims to provide a well-designed investigation into the risk ratio between artificial joint replacement and subsequent autoimmune diseases, particularly connective tissue diseases.

Silicone breast implants have been implicated in triggering chronic inflammatory responses and are linked to various symptoms, including fatigue, cognitive impairment, joint pain, muscle pain, fever, dry eyes, dry mouth, and Autoimmune/Inflammatory Syndrome by Adjuvants (ASIA). An analysis comparing 24,651 silicone breast implant recipients with 98,604 matched individuals without breast implants revealed an increased risk of autoimmune diseases post-implantation, with an odds ratio of 1.45 (95% CI, 1.21-1.73)^24^. Recent integrated evidences indicated that silicone breast implants could be associated with an increased risk of developing rheumatoid arthritis, Raynaud's syndrome, and dryness. The effect size for the development of rheumatoid arthritis was particularly notable at 1.38 (95% CI, 1.06-1.80)^25^.

While our research does not permit us to definitively determine the mechanisms underlying this phenomenon, insights gleaned from previous literature offer speculative explanations. One theory posits that breast implant-related ailments may be linked to a chronic inflammatory response triggered by the release of silicone particles from the implants. However, subsequent studies have not found a statistically significant difference in antibody levels between women with ruptured implants and those with intact implants, suggesting that silicone leakage might not be the primary cause of systemic symptoms post-implantation^44^. Another hypothesis suggests that the formation of bacterial biofilms on implants leads to prolonged interactions between the host and pathogens, potentially initiating chronic inflammation and subsequent systemic autoimmune symptoms. Bacterial biofilms have been implicated in various complications associated with implanted biomaterials, including pericondylar contracture, spondylolisthesis^47^,^48^, and pain^49^.

Furthermore, allergic reactions to metals are not uncommon, with prevalence rates ranging from 10% to 15%^49^-^51^. Metal reactions are linked to implant failure as metal ions accumulate and persistently release into the tissue surrounding the implant. This accumulation causes an inflammatory reaction, ultimately resulting in implant failure. The incidence of metal sensitization is up to six times greater in people with implant failure while comparing with the general population^51^. A macrophage-mediated immune response from joint replacement was previously identified. Some patients exhibit T lymphocytes specifically targeting metal particles. The production of excessive metal fragments is associated with type IV immune response, and T cells could be highly involved in this reaction^52^. Patients with osteolytic lesions show an elevated rate of sensitization to cobalt, and tissue sections around the joint prosthesis reveal CD3-positive T cells and CD68-positive macrophages. These findings strongly suggest that early osteolysis in metal hip replacement patients is associated with delayed-type hypersensitivity to metal^53^. Activation of the immune system by forming complexes with natural proteins triggers subsequent delayed-type hypersensitivity (DTH)^54^. These abnormal inflammatory responses may be significant risk factors for the development of autoimmune diseases. Although the detailed mechanisms are still uncertain, there is a substantial body of literature on the role of metal sensitization in contributing to the onset, progression, and severity of symptoms in these diseases (e.g., RA, chronic fatigue syndrome, and dryness)^28^. We anticipate further literature to help clarify the association between metal implants and disease.

This study possesses several notable strengths. Firstly, we utilized a robust global-federated, multi-center database for data acquisition and analysis. This platform offers precise healthcare system-specific diagnostic insights based on electronic medical records. Secondly, via conducting subgroup and sensitivity analyses, the evidences of the observed association could be further evaluated and solidified. Thirdly, we implemented robust control and adjustment measures for baseline and potential confounding factors, enabling more dependable conclusions from propensity-matched analyses conducted on substantial sample sizes. Lastly, to the best of our knowledge, this represents the pioneering study to employ a large-scale database for the analysis of the association between TKA and the risk of RA.

Inherent limitations are present in this retrospective study, stemming from its design and data source. First, our reliance on ICD-10-CM codes for patient identification and outcome assessment introduces the potential for misdiagnosis and misclassification. However, it is noteworthy that our study demonstrated a high degree of accuracy in identifying RA patients using ICD-10-CM codes, as supported by previous research^55^. Second, the severity of osteoarthritis may differ between the TKA and non TKA cohort as those who underwent TKA are likely to have a more severe disease. Nonetheless, in the current study, severity of osteoarthritis was also not able to be precisely stratified due to lacking related information. Third, despite adjustment through matching exposed and control populations, the possibility of residual confounding factors persists. Forth, the majority of participants were Americans, encompassing various ethnicities, yet the generalizability of our conclusions to Asian or European populations remains uncertain. Fifth, lack of information on the material composition of knee prostheses precludes an analysis of RA risk based on different materials. Lastly, the intricate mechanisms underlying the increased risk of RA after TKA remain undisclosed by our study alone, highlighting the need for further research to unravel this observed association.

In conclusion, our investigation has unveiled a previously uncharted connection between TKA and RA. While our discoveries offer valuable insights, additional prospective studies and in-depth mechanistic inquiries are imperative to substantiate and enhance our comprehension. This deeper understanding promises to propel our knowledge of autoimmune responses post-surgical interventions, ultimately fostering the development of more precise preventive and therapeutic strategies within the dynamic domain of joint arthroplasty.

## Supplementary Material

Supplementary tables.

## Figures and Tables

**Table 1:** Baseline characteristics of study subjects (before and after propensity score matching)

	Before matching				After matching^a^		
	TKA cohort (n=43,602)	Control cohort (n=2,043,160)	Standardized difference		TKA cohort (n=43,601)	Control cohort (n=43,601)	Standardized difference
**Age at index**							
Mean±SD	64.6±10.3	61.0±14.7	**0.28**		64.6±10.3	64.6±11.0	0.00
**Sex**							
Male	17096(39.2)	800052(39.2)	0.00		17096(39.2)	17212(39.5)	0.01
Female	26258(60.2)	1163594(57.0)	0.07		26257(60.2)	26162(60.0)	0.00
**Race, n (%)**							
White	32064(73.5)	1375618(67.3)	**0.14**		32063(73.5)	32224(73.9)	0.01
Black or African American	4558(10.5)	255173(12.5)	0.06		4558(10.5)	4511(10.3)	0.00
Asian	1180(2.7)	45086(2.2)	0.03		1180(2.7)	1183(2.7)	0.00
Native Hawaiian or other Pacific Islander	373(0.9)	7450(0.4)	0.06		373(0.9)	419(1.0)	0.01
**Lifestyle**							
Alcohol dependence, smoking and substance use	2274(5.2)	124172(6.1)	0.04		2274(5.2)	2050(4.7)	0.02
**Comorbidities**							
Hypertension	12093(27.7)	452515(22.1)	**0.13**		12092(27.7)	11763(27.0)	0.02
Diabetes mellitus	4453(10.2)	195589(9.6)	0.02		4453(10.2)	4288(9.8)	0.01
Hyperlipidemia	7191(16.5)	270172(13.2)	0.09		7190(16.5)	6953(15.9)	0.01
Ischemic heart diseases	3076(7.1)	122801(6.0)	0.04		3076(7.1)	2850(6.5)	0.02
Cerebrovascular diseases	1440(3.3)	66269(3.2)	0.00		1440(3.3)	1282(2.9)	0.02
Vitamin D deficiency	1470(3.4)	75566(3.7)	0.02		1470(3.4)	1307(3.0)	0.02
Chronic kidney disease	1084(2.5)	53806(2.6)	0.01		1084(2.5)	840(1.9)	0.04
**Socioeconomic Status**							
Health hazards related to socioeconomic and psychosocial circumstances	204(0.5)	13797(0.7)	0.03		204(0.5)	163(0.4)	0.01
**Medications**							
Antidepressants	6202(14.2)	216859(10.6)	**0.11**		6202(14.2)	5963(13.7)	0.02
Glucocorticoids	12180(27.9)	308489(15.1)	**0.32**		12179(27.9)	12100(27.8)	0.00
Beta blockers	7584(17.4)	217008(10.6)	**0.20**		7583(17.4)	7334(16.8)	0.02
**Medical Utilization Status**							
Ambulatory visit	28787(66.0)	1148428(56.2)	**0.20**		28786(66.0)	28526(65.4)	0.01
Inpatient visit	11043(25.3)	289312(14.2)	**0.28**		11042(25.3)	10876(24.9)	0.01
**Laboratory data**							
BMI, n (%)							
≥ 35 (kg/m^2^)	3823(8.8)	90924(4.5)	**0.17**		3822(8.8)	3602(8.3)	0.02
C reactive protein, n (%)							
≥ 3 (mg/L)	2982(6.8)	58926(2.9)	**0.18**		2981(6.8)	2608(6.0)	0.03
Rheumatoid factor, n (%)							
≥ 14 (IU/mL)	208(0.5)	4628(0.2)	0.04		208(0.5)	150(0.3)	0.02
Antinuclear antibody, n (%)							
≥ 1:80 (titer)	18(0.0)	450(0.0)	0.01		18(0.0)	11(0.0)	0.01
Anti Cyclic citrullinated peptide, n (%)							
≥ 20 (arbitrary unit/mL)	10(0.0)	106(0.0)	0.01		10(0.0)	10(0.0)	0.00
Erythrocyte sedimentation rate							
≥ 20 (mm/hour)	3300(7.6)	64546(3.2)	0.20		3300(7.6)	2351(5.4)	0.09

Bold font represents a standardized difference was more than 0.1; In TriNetX research platform, if the amount of population in specific item is less than or equal to 10 people, the data will be presented as 10 to ensure full de-identification. ^a^ Propensity score matching was performed on age at index, sex, race, body mass index, CRP level, status of comorbidities, comedication use, smoking, alcoholism and substance use, socioeconomic issues, medical utilization status.

**Table 2:** Risk of rheumatoid arthritis under different follow-up time^a^

Outcomes	Hazard ratio (95% Confidence interval)^b^		
	1 year	3 years	5 years
Rheumatoid arthritis	**1.74 (1.39,2.18)**	**1.31 (1.15,1.50)**	**1.33 (1.19,1.48)**

^a^Data present here were the value of follow up from 90 days after index date to the respective following up years. ^b^ Propensity score matching was performed on age at index, sex, race, body mass index, CRP level, status of comorbidities, comedication use, smoking, alcoholism and substance use, socioeconomic issues, medical utilization status.

**Table 3:** Sensitivity analysis: risk of rheumatoid arthritis in total knee arthroplasty patients based on different covariate matching models, with 5-year follow up

Outcomes	Hazard ratio (95% Confidence interval)			
	Crude	Model 1^a^	Model 2^b^	Model 3^c^
Rheumatoid arthritis	**1.32 (1.22,1.43)**	**1.25 (1.11,1.41)**	**1.23 (1.10,1.38)**	**1.25 (1.11,1.40)**

^a^ Propensity score matching was performed on age at index and sex ^b^ Propensity score matching was performed on age, sex and comorbidities ^c^ Propensity score matching was performed on age, sex, substance use and comedications

**Table 4:** Stratification analysis of rheumatoid arthritis in total knee arthroplasty patients

	Cases occurring new-onset rheumatoid arthritis		
Subgroups	Total knee arthroplasty cohort (No. of event/ Total knee arthroplasty patient amount in each subgroup)	Control cohort (No. of event/ non-total knee arthroplasty patient amount in each subgroup)	HR (95% CI)^a^
**Gender**			
Male	170/14770	134/14770	1.24 (0.99,1.55)
Female	493/22739	339/22739	**1.42 (1.24,1.63)**
**Age at index date**			
18-64 years old	118/5719	79/5719	**1.48 (1.11,1.97)**
≥ 65 years old	547/31844	387/31844	**1.38 (1.21,1.58)**

^a^ Propensity score matching was performed on age at index, sex, race, body mass index, CRP level, status of comorbidities, comedication use, smoking, alcoholism and substance use, socioeconomic issues, medical utilization status.
