# Supplementary material for: Increased Risk of New-Onset Rheumatoid Arthritis Among Osteoarthritis Patients Received Total Knee Arthroplasty: a global federated health network analysis
Source: Int J Med Sci. 2024 Apr 8;21(6):994–1002. doi: 10.7150/ijms.93457 (PMC11103392; doi:10.7150/ijms.93457)
Supplement: Supplementary file 1 — Supplementary tables. [file ijmsv21p0994s1.pdf]

Supplementary Files

Supplementary Tables

Table S1. Utilized administrative codes

**Description**	**ICD-10-CM codes** [a]
Osteoarthritis	M15-M19
Neoplasms	C00-D49
Rheumatoid arthritis	M05-M06
Diabetes mellitus	E08-E13
Hypertension	I10
Ischemic heart diseases	I20-I25
Cerebrovascular diseases	I60-I69
Hyperlipidemia	E78.5
Vitamin D deficiency	E55
Chronic kidney disease	N18
Ankylosing spondylitis	M45
Dermatopolymyositis	M33
Sjögren syndrome	M35.0
Systemic lupus erythematosus	M32
Psoriasis	L40
Ulcerative colitis	K51
Crohn's disease	K50
Health hazards related to socioeconomic and psychosocial	Z55-Z65

circumstances	
Substance abuse	F10-F19
Encounter for general examination	Z00
**Medications**	**VA codes**
Antidepressants	CN600
Glucocorticoids	HS051
Beta blockers	CV100
**Procedures**	ICD-10-PCS[b]
Total knee arthroplasty	0SRD0JZ, 0SRC0JZ, 0SRW0JZ, 0SRV0JZ, 0SRU0JZ, 0SRT0JZ, 0SRD07Z, 0SRD0KZ, 0SRU07Z, 0SRU0KZ, 0SRW07Z, 0SRW0KZ, 0SRC0KZ, 0SRT07Z, 0SRT0KZ, 0SRV0KZ

[a]ICD-10-CM: International Classification of Diseases, Tenth Revision, Clinical Modification

[b]ICD-10-PCS: International Classification of Diseases, Tenth Revision, Procedure Coding System

Table S2. Overall incidence of rheumatoid arthritis in TKA group and in non-TKA group with osteoarthritis[a]

Groups [b]	Incidence Proportion	Incidence Rate (cases/person-day)
TKA group	3.101%	0.000008
Non-TKA Control group	2.037%	0.000006

TKA: Total knee arthroplasty

[a] Time window: from 2005-01-01 to 2023-12-31

[b] In both TKA and non-TKA control group in this analysis, the incident rheumatoid arthritis was not limited as new-onset rheumatoid arthritis. Hence the history of rheumatoid arthritis has not been set as exclusion criteria in both groups.

Table S3. Sensitivity analysis: risk of rheumatoid arthritis in total knee arthroplasty patients with different wash-out periods [a,b]

Outcome	Hazard ratio (95% Confidence interval)[b]		
	12 months	24 months	36 months
Rheumatoid arthritis	**1.30 (1.15,1.47)**	**1.39 (1.21,1.59)**	**1.46 (1.26,1.70)**

[a] Incident events occurred within each wash-out period were excluded in the corresponding analysis, following up for 5 years after index date

[b] Propensity score matching was performed on age at index, sex, race, body mass index, CRP level, status of comorbidities, comedication use, smoking, alcoholism and substance use, socioeconomic issues, medical utilization status.

Table S4. Risk of rheumatoid arthritis under different follow-up time, comparing with general population[a]

Outcome	Hazard ratio (95% Confidence interval)[b]		
	1 year	3 years	5 years
Rheumatoid arthritis	**2.76 (2.13,3.58)**	**2.01 (1.73,2.34)**	**1.87 (1.66,2.11)**

[a]Data present here were the value of follow up from 90 days after index date to the respective following up years. General population refers to people with visit record and underwent general examinations in collaborative healthcare organizations in the TriNetX research network.

[b] Propensity score matching was performed on age at index, sex, race, body mass index, CRP level, status of comorbidities, comedication use, smoking, alcoholism and substance use, socioeconomic issues, medical utilization status.
